# *Oxytropis shennongjiaensis* (Fabaceae), a new species from Hubei, Central China

**DOI:** 10.3897/phytokeys.149.49533

**Published:** 2020-06-04

**Authors:** Jun-Tong Chen, Dai-Gui Zhang, Zhen-Yu Lv, Xian-Han Huang, Peng-Ju Liu, Jia-Ning Yang, Jing-Yuan Yang, Komiljon Tojibaev, Tao Deng, Hang Sun

**Affiliations:** 1 CAS Key Laboratory for Plant Diversity and Biogeography of East Asia, Kunming Institute of Botany, Chinese Academy of Sciences, Kunming 650201, Yunnan, China Kunming Institute of Botany, Chinese Academy of Sciences Yunnan China; 2 University of Chinese Academy of Sciences, Beijing 100049, China University of Chinese Academy of Sciences Beijing China; 3 Key Laboratory of Plant Resources Conservation and Utilization, Jishou University, Jishou 416000, Hunan, China Jishou University Hunan China; 4 School of Life Sciences, Yunnan Normal University, Kunming 650092, Yunnan, China Yunnan Normal University Yunnan China; 5 Administration of Shennongjia National Park, Shennongjia 442421, Hubei, China Administration of Shennongjia National Park Hubei China; 6 Central Herbarium of Uzbekistan, Institute of Botany, Academy Sciences of Uzbekistan, Tashkent 100025, Uzbekistan Institute of Botany, Academy Sciences of Uzbekistan Tashkent Uzbekistan

**Keywords:** Shennongjia National Park, phylogeny, new species, *Oxytropis shennongjiaensis*

## Abstract

Here we describe *Oxytropis shennongjiaensis*, a new species of Fabaceae from Central China (Hubei Province). Morphologically, *O. shennongjiaensis* is closely similar to *O. sitaipaiensis*, *O. melanocalyx* and *O. kansuensis*, but differs in stem characters, with less conspicuous internodes; persistent herbaceous stipules; pale yellow to white corolla; and stipitate legumes, 3–5 mm with a long beak. Phylogenetic analysis, based on the internal transcribed spacers (ITS) and two chloroplast markers (*trnL–F* and *psbA–trnH*), also identified *O. shennongjiaensis* as a new species, which is consistent with our morphological analyses. Considering the morphological data and phylogenetic data presented here, we believe that this evidence satisfies the required diagnostic criteria to identify *O. shennongjiaensis* as a new species.

## Introduction

About 310 species of *Oxytropis* DC. have been described, mainly distributed in East and Central Asia, as well as Europe, Africa and North America (Zhu et al. 2010). Zhang (1998) recorded 146 species of *Oxytropis* (incorporating 12 varieties) as native to China. However, in the Flora of China, Zhu et al. (2010) only recognised 133 species of *Oxytropis* after having eliminated taxa of uncertain taxonomic status and those based on specimen misidentifications. *Oxytropis* in China is mainly distributed in Xinjiang, Tibet, Qinghai, northwest Yunnan, western Sichuan, Gansu, Inner Mongolia, Shaanxi, Shanxi, Henan, Hebei, Liaoning, Jilin and Heilongjiang Provinces.

China has a vast territory with a wide range of complex and diverse topographies and soils and covering several climate zones, which contribute to the wealth of Chinese botanical diversity (Chen et al. 2018a). The Shennongjia National Park in Hubei Province is a world-famous natural heritage site for biodiversity richness and, in recent years, many new species have been described from the region (Chen et al. 2018b; Deng et al. 2018). In 2016, during a comprehensive collecting expedition within this Park, we discovered a species of *Oxytropis* that was very unusual in its morphological characters. After consulting local floras (Fu 1979; Qian 1990; Yang et al. 2009; Li and Liu 2010) and newly published species (Zhu and Ohashi 2000; Zhu et al. 2002; Zhu 2003), especially from the vicinity of the Park (Hubei, Anhui, Jiangxi, Hunan, Guizhou and Chongqing), we were unable to find any record of *Oxytropis* in these regions. However, there are eight species of *Oxytropis* recorded in the neighbouring Henan Province (Ding and Wang 1988). Additionally, in the neighbouring Shaanxi Province, which has the closest geographical connection, nine species and two varieties of *Oxytropis* are recorded in Flora Tsinlingensis (Northwest Institute of Botany, Chinese Academy of Sciences 1981).

After three years of observations of wild living plants, herbarium specimens and laboratory studies, we determined that the morphological characters of this entity were stable and did not match with any other species of *Oxytropis* known to us. Accordingly, combined with a molecular phylogenetic analysis, based on the internal transcribed spacers (ITS) and two chloroplast markers *trnL–F* and *psbA–trnH*, we determined that this entity was indeed a species new to science and, therefore, we describe it below as *O. shennongjiaensis* D.G. Zhang, J.T. Chen, T. Deng & H. Sun, sp. nov. As *Oxytropis* was first discovered in the mountains of Central China (Hubei Province), this new species is particularly valuable for further study of the origins, dispersal and current geographical distribution of the genus.

## Materials and methods

### Morphology

The specimens of *Oxytropis shennongjiaensis* were collected from Shennongjia National Park in Hubei Province. Morphological characters, recorded for the new species, were based on fresh ﬂowering and fruiting material. Morphological comparisons of *O. shennongjiaensis*, with related taxa *O. sitaipaiensis* T. P. Wang ex C. W. Chang, *O. melanocalyx* Bunge and *O. kansuensis* Bunge, are provided in Table 1.

**Table 1.** Morphological comparisons of *Oxytropis shennongjiaensis* with related species.

**Characters**	***O. shennongjiaensis***	***O. sitaipaiensis***	***O. melanocalyx***	***O. kansuensis***
**Plant height**	10–15 cm tall	10–13 cm tall	5–17 cm tall	12–40(–60) cm tall
**Branches**	Stems with less conspicuous internodes, 3–15 cm long.	Stems with 2 or more conspicuous internodes.	Stems with (0 or)1–4 conspicuous internodes.	Stems with (3 or)4 or 5 conspicuous internodes.
**Stipules**	Stipules ovate, 7–10 mm long, herbaceous and margin scarious.	Stipules narrowly triangular, 3–5 mm long, membranous.	Stipules ovate-triangular, herbaceous.	Stipules narrowly triangular, 5 mm long, herbaceous.
**Leaves**	Leaves with sparsely subappressed white trichomes.	Leaves with sparsely white trichomes.	Leaves with sparse yellow, white and black long trichomes.	Leaves with glabrescent or sparsely spreading white villous.
**Racemes**	Racemes rather lax, 3–6-flowered; peduncle 2.5–4.5 cm long.	Racemes rather lax, 3–5-flowered; peduncle 5–6 cm long.	Racemes compact, 3–10(–15)-flowered; peduncle 5.5–14 cm long.	Racemes compact, 3–15-flowered; peduncle 7–21(–30) cm long.
**Bracts**	Bracts ovate, 6–8 mm long, membranous.	Bracts subulate, ca. 2 mm long, membranous.	Bracts longer than pedicels, membranous.	Bracts triangular, 6–7 mm long, membranous.
**Calyx**	Calyx 9–11 × 2–4 mm; lobes subulate, 4–5 mm long.	Calyx ca. 4 × 3 mm; lobes linear, 2–3 mm long.	Calyx ca. 4–9 × 2–3.5 mm; lobes lanceolate-linear, 2.5–4.7 mm long.	Calyx 6.5–11.5 × 2–4 mm, lobes subulate, 2–8 mm long.
**Flowers**	Corolla pale yellow to white; standard 16–18 mm long, lamina broadly ovate, 12–13 × 10–11 mm, apex emarginate to 2-lobed, margin lightly undulately entire or with irregular repand teeth; wings 12–15 mm long, lamina obovate; keel 15 mm long, beak 3 mm long.	Corolla purplish; standard ca. 11 × 3 mm, lamina elliptic; wings ca. 10 mm long, lamina oblong; keel ca. 9.5 mm long, beak ca. 1.5 mm long.	Corolla blue; standard 10 × 14 mm, lamina broadly ovate, apex rounded to 2-lobed; wings 7–11 mm long, apex rounded to emarginate; keel ca. 7–11 mm long, beak ca. 0.2–1.1 mm long.	Corolla yellow or pale yellow; standard 10–17 mm long, lamina ovate, apex emarginate; wings 8–15 mm long, lamina obovate; keel 8–13 mm long, beak 0.2–1 mm long.
**Legume**	Legume stipitate; stipe 5–7 mm long; body 20–25 × 5–7 mm, erect, inflated and slightly flattened, sparsely white trichomes; beak 3–5 mm long.	Legume stipitate; stipe ca. 7 mm; body ca. 23 × 4 mm, inflated and slightly flattened, with dense white short trichomes; beak 3 mm long.	Legume sessile or with a stipe; body 15–20 × 7–12 mm, pendulous, inflated, with long trichomes.	Legume shortly stipitate; stipe 1–1.5 mm; body 8–12 × 3–10.5 mm, inflated.
**Distribution**	Hubei (Shennongjia National Park)	Shaanxi	Gansu, Qinghai, Shaanxi, Sichuan, Xinjiang, Xizang, Yunnan.	Gansu, Qinghai, Sichuan, Xizang

### Molecular analyses

Molecular analysis was performed, based on 35 samples from 34 species (incorporating one variety) belonging to 11 sections of *Oxytropis* and, as such, represents the most comprehensive phylogeny of Chinese *Oxytropis* undertaken to date. *Astragalus daenensis daenensis*Boissier and *A. penetratus* Maassoumi were chosen as outgroups, following Shahi-Shavvon et al. (2017). Sequences for 34 related *Oxytropis* taxa and the two outgroup taxa were obtained from the NCBI GenBank. The GenBank accession numbers are listed in Appendix I. DNA of *O. shennongjiaensis* was isolated using a Plant Genomic DNA Kit DP305 (Beijing, China), for use as template in subsequent Polymerase Chain Reactions. Based on earlier studies, we chose ITS and two chloroplast DNA sequences (*trnL–F* and *psbA–trnH*) to perform the phylogenetic analysis (Shahi Shavvon et al. 2017; Lu et al. 2010; Li et al. 2011). Sequences were assembled and a multiple alignment was initially performed using MAFTT in Geneious version 9.0.2 (Kearse et al. 2012), followed by minor manual corrections. Gaps were treated as missing data.

Phylogenetic relationships were assessed using Bayesian Inference (BI) analyses, maximum parsimony (MP) and maximum likelihood (ML). A MP phylogenetic tree was constructed using PAUP* version 4.0a (Swofford 2002). The heuristic search was selected using 1000 replicates of random addition sequence and tree bisection-reconnection (TBR). Branch support was evaluated by 1000 bootstrap values. The ML phylogenetic tree was conducted in the IQ-TREE webserver (Trifinopoulos et al. 2016, http://iqtree.cibiv.univie.ac.at). Substitution model options were set to Auto and analysis, followed by 1,000 replicates. BI analyses were calculated in MrBayes version 3.2.7 (Ronquist and Huelsenbeck 2003). Models of sequence evolution for each partition were determined following the Akaike Information Criterion (AIC), as implemented in jModelTest, version 2.1.6 (Posada 2008). The results showed that the TIM3ef+I model was identified as the best-fit for ITS, the TIM1+I model for *psbA–trnH* and the TIM2+I model for *trnL–F*. These models cannot be found in MrBayes and GTR+I was thus selected as a replacement. Bayesian analyses were done using the settings: Bayesian trees were started from random trees; four Markov Chain Monte Carlo (MCMC) simulations were run simultaneously and sampled every 1,000 generations for a total of 10 million generations; and the first 20% of trees were discarded as burn-in.

## Results

### Taxonomic treatment

#### Oxytropis shennongjiaensis

Taxon classificationPlantaeFabalesFabaceae

D.G. Zhang, J.T. Chen, T. Deng & H. Sun sp. nov.

1839F096-BFB7-5EC1-AC8A-D52B88F9D469

urn:lsid:ipni.org:names:77209856-1

Figures 1 , 2 , 3

##### Type.

China. Hubei: Shennongjia National Park, 31°26'39.96"N, 110°16'00.34"E, 2880 m elev., 9 June 2019, *D.G. Zhang* & *Q. Liu* 19060901 (holo: KUN barcode 1347953!; iso: JIU!).

##### Diagnosis.

Compared with the published species of *Oxytropis* in China, *O. shennongjiaensis* appears to be closely similar to *O. sitaipaiensis*, from which it can be distinguished by its stems with less conspicuous internodes and 5–15 mm internodes (stems with two or more conspicuous internodes in *O. sitaipaiensis*); stipules ovate, 7–10 mm long, herbaceous (stipules narrowly triangular, 3–5 mm long, membranous in *O. sitaipaiensis*); bracts ovate, 6–8 mm long (bracts subulate, ca. 2 mm long in *O. sitaipaiensis*); calyx 9–11 × 2–4 mm (calyx ca. 4 × 3 mm in *O. sitaipaiensis*); pale yellow to white corolla; beak 3 mm long (purplish corolla; beak ca. 1.5 mm long in *O. sitaipaiensis*). Table 1 provides detailed morphological comparisons with similar species.

**Figure 1.**
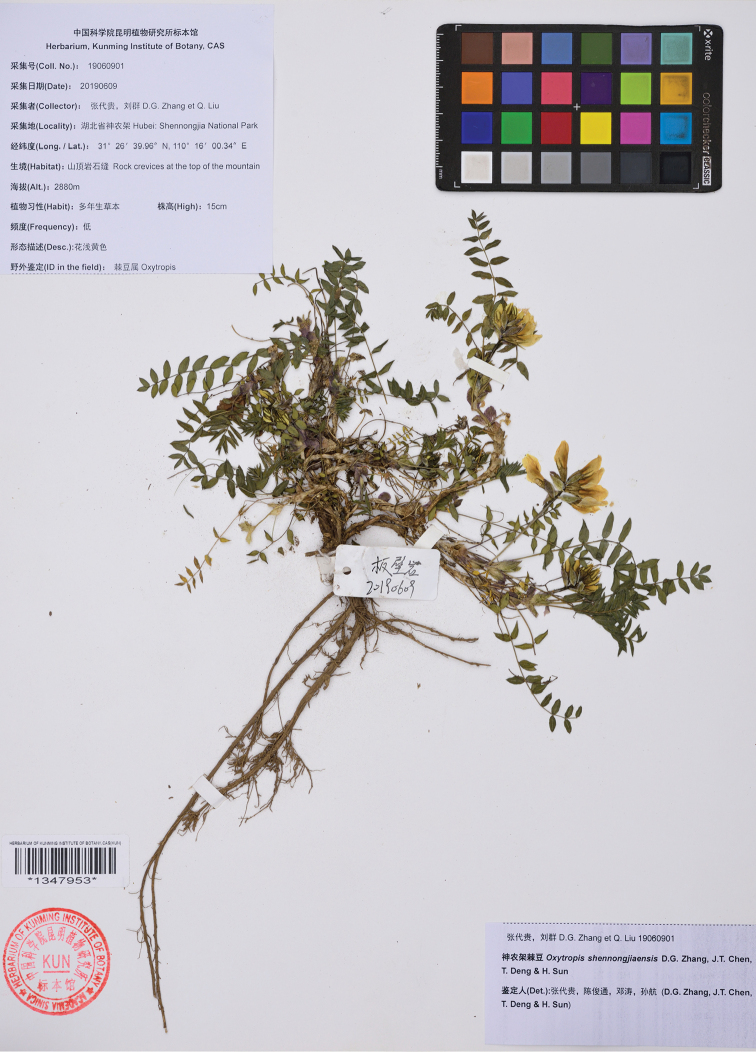
Photograph of the holotype of *Oxytropis shennongjiaensis* D.G. Zhang, J.T. Chen, T. Deng & H. Sun (KUN barcode 1347953).

##### Description.

Perennial herbs, 10–15 cm tall. Yellowish-brown, cylindrical roots, up to 25 cm long, with lateral roots. Caulescent from a multi-headed caudex, slightly subterranean sometimes rhizomatous. Stems sprawling, 3–15 cm long, basally with persistent stipules; nodes of stems slightly swollen; internodes 5–15 mm long, invested with sparse, white trichomes. Leaves (4–) 6–9 (–11) cm long, 13–17 (–19)-foliolate; leaflets ovate, 5–11 × 2–4 mm, apex acuminate, with sparse, subappressed white trichomes, abaxially mid-vein slightly raised (obvious after drying), with denser trichomes along vein; dark purplish-red or green rachis, with sparse white trichomes; stipules ovate, 7–10 × 3–4 mm, herbaceous, basally connate, apex acuminate, abaxially sparsely hairy with white trichomes, adaxially glabrous, margins scarious, ciliate with black and white trichomes. Racemes rather lax, 3–6-flowered; peduncles 2.5–4.5 cm long, erect, villous, with white trichomes, sparsely intermixed with black trichomes below, with densely black trichomes above. Bracts ovate, 6–8 × 2–3 mm, membranous, with sparse, dark brown trichomes intermixed with white trichomes abaxially. Calyx campanulate, 9–11 × 2–4 mm, with dark brown trichomes sparsely intermixed with white trichomes outside; lobes subulate, 4–5 mm long, as long as or sometimes slightly shorter than tube. Pale yellow to white corolla; standard 16–18 mm long, lamina broadly ovate, 12–13 × 10–11 mm, apex emarginate to 2-lobed, margins slightly undulated entire or with irregular repand teeth; wings 12–15 mm, lamina obovate, 7 × 4 mm long, apex obtuse, claw 4–5 mm long; keel 15 mm long, beak 3 mm long. Ovary linear, with dense white trichomes. Legumes stipitate (stipe 5–7 mm long), oblong-ellipsoid, 20–25 × 5–7 mm, erect, inflated and slightly flattened, with sparsely white trichomes, beak 3–5 mm long.

**Figure 2.**
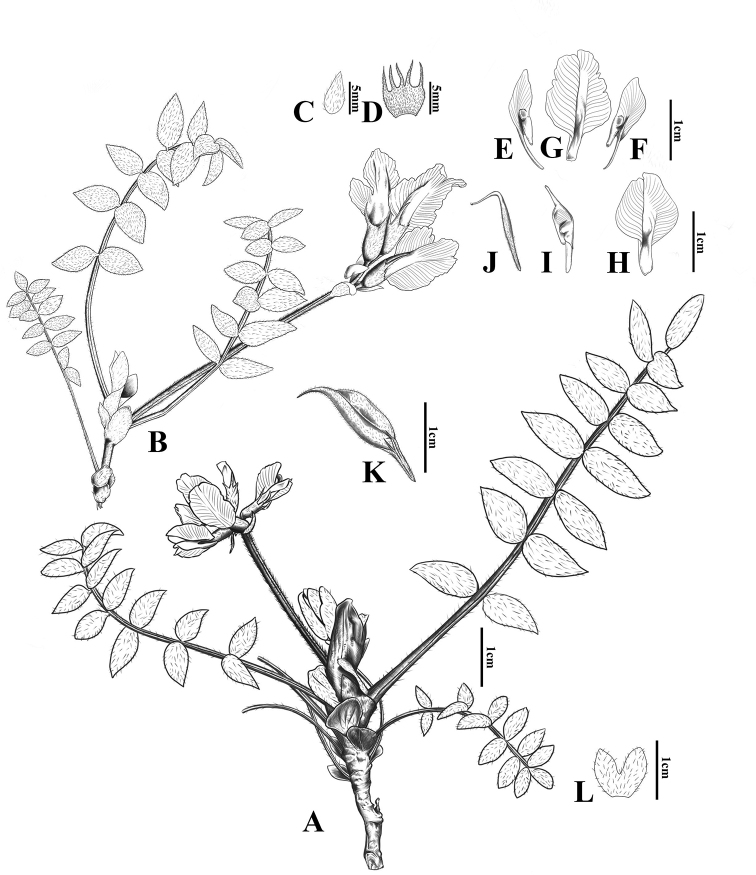
*Oxytropis shennongjiaensis* D.G. Zhang, J.T. Chen, T. Deng & H. Sun **A, B** plant showing flowering branch and leaves **C** bract **D** calyx **E–F** wing **G–H** standard (view from inside) **I** keel **J** ovary **K** legume **L** stipules. (Drawn based on the holotype of D.G. Zhang & Q. Liu 19060901 by J. N. Yang).

##### Phenology.

Flowering from May–June and fruiting from July–August.

##### Etymology.

The specific epithet refers to the Shennongjia National Park to which the species is endemic. The Chinese name is 神农架棘豆, shén nóng jià jí dòu in Chinese phonetic transcription.

**Figure 3.**
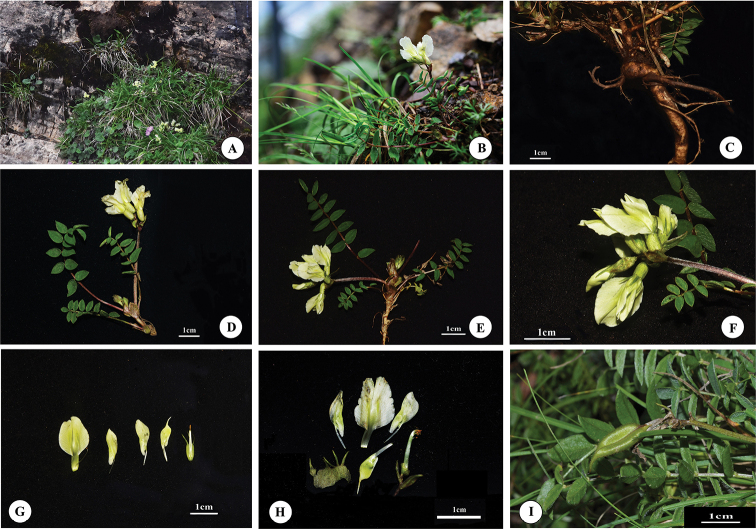
*Oxytropis shennongjiaensis* D.G. Zhang, J.T. Chen, T. Deng & H. Sun **A, B** habitat **C** root **D, E** flowering branch and leaves **F** raceme (close-up) **G, H** floral parts (showing calyx, standard, wings, keel and stamens) **I** legume.

##### Distribution and habitat.

**Figure 4.**
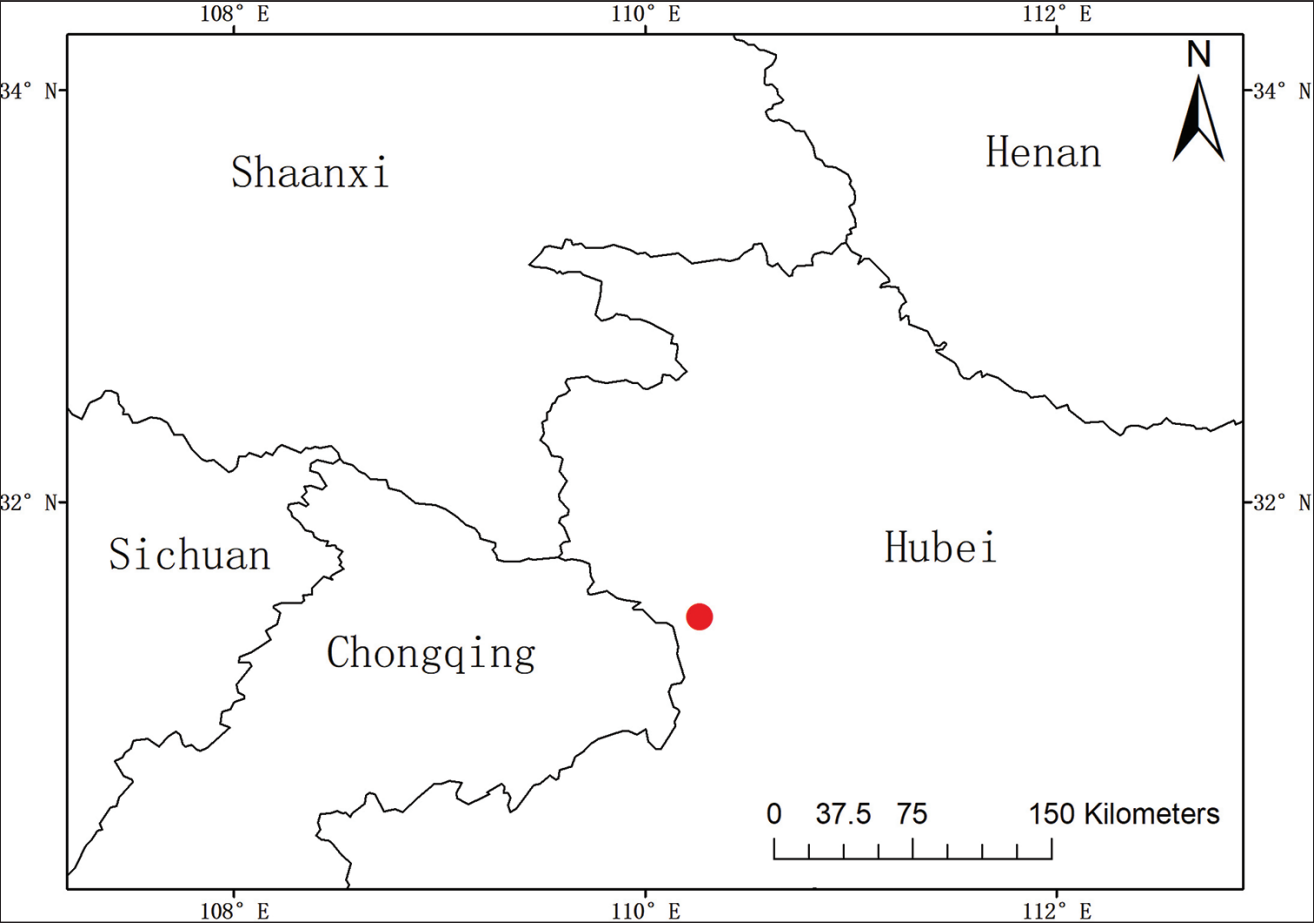
Known distribution of *Oxytropis shennongjiaensis* D.G. Zhang, J.T. Chen, T. Deng & H. Sun (The red dot represents the distribution site).

The new species is currently known only from the Shennongjia National Park (Figure 4), Hubei, China, at an elevation of 2,880 m. It grows in barren rock crevices at the top of a mountain together with *Polygonum macrophyllum* D.Don (Polygonaceae), *Primula* sp. (Primulaceae), *Carex* sp. (Cyperaceae), *Chrysanthemum oreastrum* Hance (Asteraceae), *Dracocephalum rupestre* Hance (Lamiaceae) and *Meconopsis quintuplinervia* Regel (Papaveraceae) etc.

##### Conservation status.

The new species was only discovered in Jinsiyanya, Shennongjia National Park, from our expeditions during the past few years. About 300 individuals were observed and the extent of occurrence is ca. 50,000 m^2^. The precise conservation status of the population(s) has not been determined, so further explorations are needed to assess its conservation status. Based on available data, the new species is assigned to the category ‘Data Deficient’ (DD) of International Union for Conservation of Nature (IUCN 2019).

##### Molecular phylogenetic analysis.

**Figure 5.**
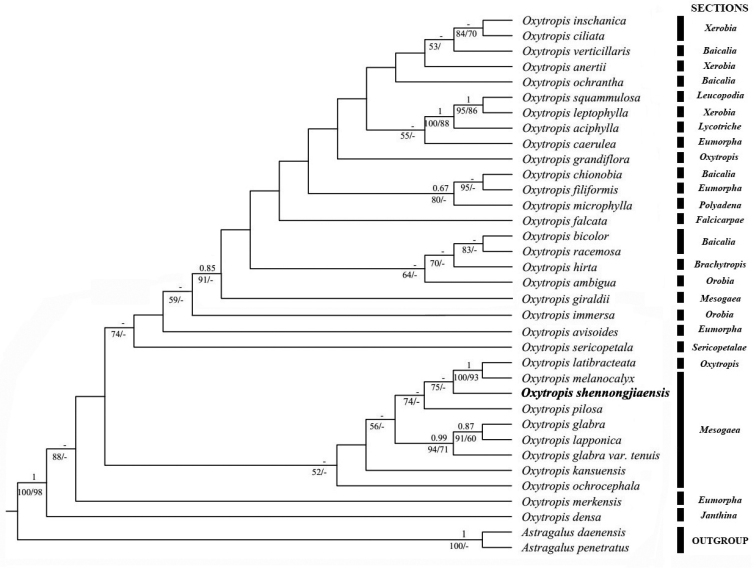
Maximum likelihood consensus tree of *Oxytropis shennongjiaensis* and related taxa. Numbers above branches indicate Bayesian posterior probability [PP], numbers below branches represent maximum likelihood bootstrap support [ML/BS] and maximum parsimony bootstrap support [MP/BS] values. Only bootstrap values > 50% are shown. The new species is shown in bold.

Based on the combined datasets (ITS, *trnL–F* and *psbA–trnH*), BI, MP and ML trees were reconstructed and their topologies are quite similar. The ML tree is presented in Figure 5 and shows the posterior probability (PP), ML bootstrap support (MLBS) and MP bootstrap support (MPBS) values. Our phylogenetic analyses show *Oxytropis shennongjiaensis* to be nested within a polyphyletic Sect.Mesogaea Bunge. *O. melanocalyx* (Sect.Mesogaea Bunge) and *O. latibracteata* (Sect.Oxytropis Bunge) are shown to be sister to *O. shennongjiaensis*, with relatively high support (ML/BS = 75). This new species is shown to be separated from other species and, to some extent, it can be identified as a new species.

## Discussion

These above-detailed characters indicate that, according to Zhang (1998), this new species belongs to Oxytropis sect.Oxytropis with 19 other species (incorporating one variety) and, according to Zhu et al. (2010), it belongs to the Oxytropis sect.Mesogaea Bunge with 32 other species (incorporating three varieties). It can be distinguished from all other species of these two sections in branches, leaves, racemes, flowers and legumes characters, as described above. Morphologically, the new species shows some similarities with *Oxytropis sitaipaiensis*, *O. melanocalyx* and *O. kansuensis* and we also examined the specimens of these species (Zhu et al. 2000), but they are also easily distinguished (Table 1). Additionally, there is no previous record of this genus in Hubei Province.

Phylogenetic analyses, based on 35 samples from 34 species (incorporating one variety), show that their topologies of the BI, MP and ML trees were quite similar and were consistent with previous studies (Shahi-Shavvon et al. 2017). *Oxytropis* is a monophyletic group. However, partial PP and BS of the tree were relatively low, which might be caused by the rapid radiation of *Oxytropis* (Shahi-Shavvon et al. 2017) and phylogenetic relationships of the new species and *Oxytropis* require further study. *O. melanocalyx* and *O. latibracteata* were shown to be sister to *O. shennongjiaensis*. These species share some morphological similarities. However, morphologically, *O. latibracteata* also shows the greatest differences in the following characters: acaulescent; racemes rather dense, 5–13-flowered or more; bluish-purple to pale purple corolla; standard 21–27 mm, lamina narrowly elliptic; wings 17–19 mm; keel 16–17 mm, beak 1–1.5 mm; legume sessile.

Considering the morphological data and phylogenetic results, we believe that this evidence satisfies the required diagnostic criteria to identify *O. shennongjiaensis* as a new species.

## Supplementary Material

XML Treatment for Oxytropis shennongjiaensis

## Appendix 1

**Table:** List of taxa used in the phylogenetic analysis of GenBank accession numbers (ITS / *trnL–F* / *psbA–trnH*).

**Species**	**ITS**	***trnL–F***	***psbA–trnH***
***Oxytropis shennongjiaensis***	**MT326210**	**MT325864**	**MT325865**
***Oxytropis aciphylla***	**GQ422810**	**JX878501**	**KF011559**
***Oxytropis ambigua***	–	**LN898539**	**LN898577**
***Oxytropis anertii***	**EF685971**	–	–
***Oxytropis avisoides***	**LC213314**	–	–
***Oxytropis bicolor***	**HQ199317**	–	–
***Oxytropis caerulea***	**HQ199316**	–	**GU396771**
***Oxytropis chionobia***	**LC213335**	**LC213480**	–
***Oxytropis ciliata***	**HQ199323**	**KC936889**	**KF011560**
***Oxytropis densa***	**LC213347**	**LC213486**	–
***Oxytropis falcata***	**KJ143722**	–	–
***Oxytropis filiformis***	**HQ199321**	**LN898596**	**LN898483**
***Oxytropis giraldii***	**LC213352**	**LC213491**	–
***Oxytropis glabra***	**LC213354**	**LC213492**	**LT856572**
**Oxytropis glabra var. tenuis**	**GQ422805**	**KC936891**	**KF011569**
***Oxytropis grandiflora***	**HQ199315**	–	–
***Oxytropis hirta***	**LC213363**	**LC213496**	–
***Oxytropis immersa***	**LC213366**	–	–
***Oxytropis inschanica***	**HQ199322**	**JX893502**	**KF011571**
***Oxytropis kansuensis***	**KJ143724**	–	–
***Oxytropis lapponica***	**LC213388**	–	–
***Oxytropis latibracteata***	**LC213389**	–	–
***Oxytropis leptophylla***	–	**JX893503**	**KF011572**
***Oxytropis melanocalyx***	**LC213397**	**LC213519**	–
***Oxytropis merkensis***	**LC213398**	**LC213520**	–
***Oxytropis microphylla***	**KP338205**	–	**KP338460**
***Oxytropis ochrantha***	**GQ422819**	**JX893489**	**KF011574**
***Oxytropis ochrocephala***	**LC213409**	–	–
***Oxytropis pilosa***	**KM053396**	**LN898607**	**LN898495**
***Oxytropis racemosa***	**HQ199320**	**JX893508**	**GU396818**
***Oxytropis sericopetala***	**KJ143725**	–	–
***Oxytropis squammulosa***	**HQ199318**	**JX893509**	**KF011579**
***Oxytropis verticillaris***	**GQ422815**	**JX893514**	**KF011581**
***Astragalus daenensis***	**AB051963**	–	–
***Astragalus penetratus***	**AB231100**	–	–

## References

[B1] Chen JT Zhong J Shi XJ Zhang QX Sun M (2018a) *Chrysanthemum yantaiense*, a rare new species of the Asteraceae from China. Phytotaxa 374(1): 92–96. 10.11646/phytotaxa.374.1.9

[B2] Chen YS Deng T Zhou Z Sun H (2018b) Is the east Asian flora ancient or not? National Science Review 5(6): 142–154. 10.1093/nsr/nwx156

[B3] Deng T Zhang DG Sun H (2018) Flora of Shennongjia (Vol. 2). China Forestry Publishing House, Beijing, 301–304. [in Chinese]

[B4] Ding BZ Wang SY (1988) Flora Henanensis (Vol. 2). Henan Science and Technology Press, Zhengzhou, 374–379. [in Chinese]

[B5] Fu SX (1979) Flora Hubeiensis (Vol. 2). Hubei Science and Technology Press, Wuhan, 234–237. [in Chinese]

[B6] Kearse M Moir R Wilson A Stones-Havas S Cheung M Sturrock S Buxton S (2012) Geneious Basic: An integrated and extendable desktop software platform for the organization and analysis of sequence data. Bioinformatics 28: 1647–1649. 10.1093/bioinformatics/bts199 22543367 PMC3371832

[B7] Li BG Liu LH (2010) Flora of Hunan (Vol. 3). Hunan Science & Technology Publishing House, Changsha, 607–610. [in Chinese]

[B8] Li YX Lan FR Chang CY Guo ZK (2011) Molecular phylogeny of *Oxytropis* DC. of Qinghai-Tibetan Plateau by ITS and trnL-F sequences. Journal of Northwest A & F University (Nat. Sci. Ed.) 39(11): 187–193.

[B9] Lu P Gao J Wang JN (2010) Molecular phylogenetic analysis of several *Oxytropis* DC. species in Inner Mongolia based on 5.8SrDNA/ITS sequences. Xibei Zhiwu Xuebao 31(12): 2420–2428.

[B10] Northwest Institute of Botany, Chinese Academy of Sciences (1981) Flora Tsinlingensis (Vol. 1(3)). Science Press, Beijing, 62–69. [in Chinese]

[B11] Posada D (2008) jModelTest: phylogenetic model averaging. Molecular Biology and Evolution 25(7): 1253–1256. 10.1093/molbev/msn083 18397919

[B12] Qian XH (1990) Flora Anhweiensis (Vol. 3). China Prospect Publishing House, Beijing, 97–101. [in Chinese]

[B13] Ronquist F Huelsenbeck JP (2003) MrBayes 3: Bayesian phylogenetic inference under mixed models. Bioinformatics (Oxford, England) 19: 1572–1574. 10.1093/bioinformatics/btg180 12912839

[B14] Shahi-Shavvon R Kazempour-Osaloo S Maassoumii AA Moharrek F Karaman-Erkul S Lemmon AR Lemmon EM Michalak I (2017) Increasing phylogenetic support for explosively radiating taxa: the promise of high-throughput sequencing for *Oxytropis* (Fabaceae). Journal of Systematics and Evolution 55(4): 385–404. 10.1111/jse.12269

[B15] IUCN (2019) Guidelines for Using the IUCN Red List Categories and Criteria. version 13. Prepared by the Standards and Petitions Subcommittee of the IUCN Species Survival Commission, 113 pp. http://cmsdocs.s3.amazonaws.com/RedListGuidelines.pdf

[B16] Swofford D (2002) PAUP*: phylogenetic analysis using parsimony (*and other methods). Version 4. Sinauer Associates, Sunderland.

[B17] Trifinopoulos J Nguyen LT von Haeseler A Minh BQ (2016) W-IQ-TREE: A fast online phylogenetic tool for maximum likelihood analysis. Nucleic Acids Research 44(W1): W232–W235. 10.1093/nar/gkw256 PMC4987875 27084950

[B18] Yang CX Xiong JH Zhong SL (2009) Keys to vascular plants in Chongqing. Sichuan Science & Technology Publishing House, Chengdu, 339–345. [in Chinese]

[B19] Zhang ZW (1998) Fabaceae (5) *Oxytropis*. In: Cui HB (Eds) Flora Reipublicae Popularis Sinicae (Vol. 42(2)). Science Press, Beijing, 146 pp. [in Chinese]

[B20] Zhu XY (2003) A new species of *Oxytropis* (Baicalia) (Leguminosae) from Shanxi province in China. Nordic Journal of Botany 23(3): 279–281. 10.1111/j.1756-1051.2003.tb00394.x

[B21] Zhu XY Ohashi H (2000) Systematics of Chinese *Oxytropis* DC. (Leguminosae). Cathaya 11 & 12: 1–218.

[B22] Zhu XY Du YF Ohashi H (2000) Catalogue of the Type Specimens of *Oxytropis* DC. (Leguminosae) (1). China Science & Technology Press, Beijing, 135 pp.

[B23] Zhu XY Du YF Ohashi H (2002) A new species of *Oxytropis* (Leguminosae) from Xizang (Tibet) in China. Novon 12(3): 430–432. 10.2307/3393094

[B24] Zhu XY Welsh SL Ohashi H (2010) *Oxytropis*. In: Wu ZY Raven PH Hong DY (Eds) Flora of China (Vol.10) (Fabaceae). Science Press, Beijing & Missouri Botanical Garden Press, St. Louis, 453–500.

